# The usefulness of uterine lavage and acute phase protein levels as a diagnostic tool for subclinical endometritis in Icelandic mares

**DOI:** 10.1186/s13028-016-0233-4

**Published:** 2016-09-07

**Authors:** Monika Sikora, Jarosław Król, Marcin Nowak, Tadeusz Stefaniak, Gudmar Aubertsson, Roland Kozdrowski

**Affiliations:** 1 Department of Reproduction and Clinic of Farm Animals, Faculty of Veterinary Medicine, Wroclaw University of Environmental and Life Sciences, Plac Grunwaldzkistreet 49, 50366 Wroclaw, Poland; 2 Department of Pathology, Faculty of Veterinary Medicine, Wroclaw University of Environmental and Life Sciences, C. K. Norwidastreet 31, 50375 Wroclaw, Poland; 3 Department of Immunology, Pathophysiology and Veterinary Preventive Medicine, Faculty of Veterinary Medicine, Wroclaw University of Environmental and Life Sciences, C. K. Norwidastreet 31, 50375 Wroclaw, Poland; 4 Dýralæknir Sandhólaferja, 851 Hella, Iceland

**Keywords:** Mare, Endometritis, Efflux, Acute phase proteins

## Abstract

**Background:**

Endometritis is a common problem in a broodmare practice, often leading to infertility. The diagnosis is based on several methods such as cytology, bacteriology and histopathology; however the outcome of these methods may be inconclusive even when used together. The objectives of this study were: (1) to investigate the usefulness of acute phase proteins as an additional diagnostic tool for diagnosis of subclinical endometritis in mares and (2) to evaluate the association between macroscopic changes in uterine flushes and inflammation of the uterus. Materials were collected from 53 Icelandic mares with subclinical endometritis. Endometrial swabs and uterine lavage for cytological and bacteriological examinations and two endometrial biopsies were taken. Blood samples were collected 12–24 h after ovulation to determine the concentrations of serum amyloid A and haptoglobin in the 53 subfertile mares and, for comparison, from 20 non-pregnant mares that later conceived.

**Results:**

Twenty-five mares were classified as positive for endometritis based on endometrial biopsy, which was used as the ‘gold standard’. We observed a correlation between cloudy efflux in the lavage and (1) polymorphonuclear cell (PMN) infiltration of the endometrium (*P* = 0.031), (2) positive cytology in samples obtained by cotton swabs (*P* = 0.019) and uterine lavage (*P* = 0.011), and (3) positive microbiology from samples obtained by cotton swabs (*P* = 0.001) and uterine lavage (*P* = 0.047). The degree of agreement between PMN infiltration and positive cytology from samples taken by cotton swabs and uterine lavage was fair to moderate. We found no association between the concentration of acute phase proteins and infiltration of the endometrium by PMNs, or with positive results of cytological and microbiological examinations.

**Conclusions:**

Measurement of serum amyloid A and haptoglobin was not proven useful for diagnosis of subclinical endometritis in Icelandic mares. Macroscopic changes in the fluid collected by lavage were not consistently indicative of infection, but when present they indicate inflammation in the uterus with a high probability.

## Background

Inflammation of the endometrium is one of the main causes of reduced fertility in the mare [1]. According to published data, it is the third most important clinical problem in horses after colic and respiratory diseases [2].

Traditionally, the diagnosis of endometritis is based on history, clinical findings and results of microbiological, cytological and histopathological examinations [3]. A detailed history of infertility can provide many important clues. LeBlanc and Causey reported that a short inter-estrus interval may already indicate the presence of abnormalities in the uterus [3]. Detection of intrauterine fluid is an indicator for clinical endometritis, however this sign may be absent in subclinical cases of endometritis [3, 4]. Subclinical endometritis has been described as a ‘hidden’ endometritis in mares, which did not show the typical signs of endometritis such as accumulation of fluid in the uterus [3].

The breeding season of mares is regulated by photoperiod because lengthening of days activates their reproductive system. Conditions for the domestic maintenance of horses in Iceland are broadly very similar to the natural situation. Foaling takes place in the field and should happen in the early summer to allow foals to reach a reasonable size before winter. Therefore, the timely diagnosis and effective treatment of infertility are very important for successful breeding in Iceland.

Traditionally in Iceland, the mares are covered by a stallion in a harem, without human intervention. In recent times, artificial insemination and hand mating have become popular, allowing deeper observation of infertility in these mares; however, no report is available on the causes of infertility in Icelandic horses.

One of many methods used both for treatment of endometritis and to collect samples from the uterus for cytological and microbiological examinations is uterine lavage (UL). LeBlanc et al. [5] and Christoffersen et al. [6] used a low volume flush, infusing 60 and 250 ml of fluid, respectively, into the uterus. After centrifuging the recovered fluid, the supernatant was decanted and the pellets were used for cytological and microbiological examinations.

Endometrial biopsy is internationally recognized as a reliable, risk-free and meaningful method for diagnosing endometritis and for prognostic evaluation of endometrial conditions in the mare [7]. However, the long waiting time for test results makes it inappropriate for immediate treatments [4, 8, 9]. Routinely, in practice only one biopsy is collected, but results obtained from a single biopsy sample may be misleading. A study performed after slaughter showed that a single biopsy is not enough to determine the severity of degenerative changes in the mare uterus with sufficient accuracy [10]. Another post mortem study revealed that evaluation of inflammation using both cytological and histological techniques does not give the same results in different sampling sites [11].

In many cases, despite negative results obtained from cytology, microbiology and histopathology, the mare still could not become pregnant.

Acute phase proteins (APP) are sensitive, but not specific, systemic markers of inflammation [12]. Serum amyloid A (SAA) is a protein that increases rapidly, with larger amplitude (>100-fold) changes after tissue injury, infection or inflammation. The concentration of SAA in healthy horses is very low or undetectable and ranges from <0.5 to 20 mg/l [13, 14]. Haptoglobin (Hp) is already present in blood plasma of healthy horses (0.2–1 g/l) and its concentration increases only 1–10 times during inflammation [14]. Tuppits et al. [15] found no differences in the concentrations of APP before and after artificial insemination. However, Christoffersen et al. [16] observed a transient increase of SAA concentration after intrauterine infusion with a high dose of *Escherichia coli* during diestrus. In contrast to these results, Christoffersen et al. [1] found no changes in plasma concentrations of SAA after inoculation with lower doses of *E. coli* during estrus and these differences appear to be both dose-dependent and cycle-related. Krakowski et al. [17] suggested that high concentrations of SAA and Hp prior to ovulation and insemination may be an indicator of latent subclinical endometritis, which cannot be diagnosed by ultrasonography. This suggested to us the need to examine the usefulness of APP levels as an additional tool for diagnosing subclinical endometritis.

The aim of this study was to evaluate the usefulness of uterine lavage with 1 l physiological saline and measurement of APP as a diagnostic tool for subclinical endometritis in Icelandic mares. In this study, the concentrations of SAA and Hp in blood collected from barren and fertile mares were examined to evaluate the usefulness of this test.

## Methods

### Mares

All samples were collected from Icelandic Horse mares, in estrus, during one breeding season from May to August 2014 in Dýralæknir Sandhólaferja Veterinary Clinic, Hella in Iceland.

Our study group consisted of 53 mares, aged from 3 to 25 (12.34 ± 5.72) years, with a history of infertility. All these mares were suspected for subclinical endometritis. They were qualified for the study based on the following criteria: they had been bred three or more times unsuccessfully in the same breeding season, or had a history of a minimum of 1 year’s reproductive failure. All the mares were in estrus, ultrasound examination of the reproductive tract showed no intrauterine fluid and no discharge from the vagina was observed. All were clinically healthy, they were kept under the same conditions and none was previously treated for endometritis.

### Control group

The control group comprised 20 fertile mares of the Icelandic Horse breed, which became pregnant after insemination or natural covering. All these mares were clinically healthy, kept under the same conditions and had no history of infertility. In this group, blood samples were collected to determine the concentrations of SAA and Hp in healthy, fertile mares that subsequently became pregnant after insemination. All of the mares were in estrus, with no intrauterine fluid observed in the ultrasound examination and no discharge from the vagina. All mares were evaluated by transrectal palpation and ultrasonography at intervals of 24 h to assess the time of ovulation. Blood samples were collected 12–24 h after ovulation. Fifteen days after ovulation, a pregnancy check was performed with ultrasound. From the material collected, only plasma samples that came from mares diagnosed positively for pregnancy were used for determination of SAA and Hp. Twenty mares were diagnosed as pregnant 15 days after ovulation, and in blood plasma from these mares the concentrations of SAA and Hp were determined. The concentrations of SAA and Hp taken from 53 barren mares were compared with their levels in 20 non-pregnant mares that later conceived. This comparison was used to investigate differences in the concentrations of APP in barren mares and in healthy, fertile mares that achieved pregnancy at the first attempt.

### Sample collection

All mares were examined while restrained in stocks. The sample collections were performed after a complete reproductive assessment of the mares, including evaluation of the external genital tract and manual transrectal palpation as well ultrasonography examinations (using Ge Logiq Book XP, USA) of the reproductive tract to determine cycle stage and to exclude intrauterine fluid. During examination of the external genital tract, in all of these mares 80 % of the labia lengths were below the pelvic brim. Afterwards, the tail was bandaged, the vulva and the perineum were cleaned with povidone-iodine (Betadine®-Polyvidonum iodinatum, MEDA Pharma S.p.A., Milan, Italy) and dried with a paper towel and samples were always collected in the same order: swab, lavage and biopsies.

### Cotton swabs

First, for cytological and bacteriological examinations, material from the uterus was collected using a double-guarded uterine cotton swab (CS; Minitube, Tiefenbach, Germany). The cotton swab was inserted into the uterus by manually passing it through the cervix. The tip of the swab was hidden in the hand and the labia were spread by additional staff to minimize infection by vaginal and vulvar skin flora. The procedure for sample collection by cotton swabs was carried out as described by Walter et al. [18].

### Uterine lavage

Then, uterine lavage (UL) was performed using a commercial sterile uterus flushing tube (EQUIVET Uterine Flushing Tube Sterile; Kruuse, Denmark). The sterile catheter for UL was manually passed into the uterus and 1 l of sterile physiological saline warmed to 38 °C was infused from a bag into the uterus. The fluid was allowed to become distributed within the uterus and then collected into the same bag by gravity flow. The recovered fluid volume and color were recorded as described below and the bag was hung for sedimentation by gravity for at least 1 h. The clarity of the recovered fluid was recorded according to LeBlanc et al. [5] and was graded as clear, cloudy, efflux with debris (mucus stains) and efflux with blood (from pink to red coloration). Then, 50 ml of efflux was aspirated from the bottom of the bag, which was previously disinfected with alcohol, by puncturing it with a sterile needle into a 50 ml tube and centrifuged at ×400*g* for 10 min. The supernatant was decanted and the sediment was used for cytological and bacteriological examinations.

### Endometrial biopsies

After lavage, two endometrial biopsies (EB) were taken from the dorsal wall of the base of each uterine horn (left and right) and fixed in 10 % formalin for histopathological examination. The biopsies were collected using a sterilized biopsy punch (Equi-Vet, Kruuse, Denmark) that was manually passed through the cervix and then directed into the left and right horns with a finger and through rectal manipulation with the other hand. The biopsies were always taken in the same order: first from the left and then from the right horn. The endometrial biopsies were immediately fixed and sent to the Department of Pathology at the Wroclaw University of Environmental and Life Sciences in Poland, where histopathological examination for inflammation of the endometrium was performed.

### Blood sample collections

Blood samples were collected once from 12 to 24 h after ovulation from the external jugular vein into 10 ml heparinized tubes. From each mare, 10 ml of blood was collected into heparinized tubes and centrifuged at ×800*g* for 10 min. Then the serum was transferred to 2 ml microcentrifuge tubes, stored at −20 °C and sent on dry ice to the Department of Immunology, Pathophysiology and Veterinary Preventive Medicine at the Wroclaw University of Environmental and Life Sciences in Poland, where the concentration of APP was determined.

### Examination of samples

Cytological smears were performed on cellular material collected using cotton swabs and from a sterile swab that was placed into the sediment obtained from uterine flushing. The swabs were rolled onto a sterile microscope glass and air-dried. The smears were stained using Diff-Quick stain (Medion Diagnostics AG, Duedinden, Switzerland) and examined with light microscopy under oil immersion (1000× magnification) for the presence of polymorphonuclear cell (PMN). We counted 300 cells in each sample. When PMN cells represented more than 2 % of all cells in the sample, it was considered positive for endometritis [11, 19–21]. The samples collected by CS and UL were evaluated according to the same criteria.

Next, the same samples that had been used for cytology were placed on a blood agar (bioMérieux, France, supplemented with 5 % defibrinated sheep blood), MacConkey Agar (bioMérieux) and Sabouraud Dextrose Agar (Oxoid, UK) for microbiological examination. The media were incubated aerobically at 37 °C for 24 h and the growth of microorganisms was recorded. Negative plates were incubated further and re-examined after 48 and 72 h for the presence of bacteria and yeasts. Then the plates were stored in a refrigerator and sent chilled to the Department of Pathology at the Wroclaw University of Environmental and Life Sciences, Poland, where microbiological examination was conducted. Isolated bacteria were preliminarily identified based on colony characteristics and cell morphology, Gram staining, hemolytic activity and a catalase test. Gram-positive and catalase-positive cocci were further examined using the ID 32 STAPH identification system (bioMérieux). Gram-positive rods were identified by means of the API CORYNE kit (bioMérieux), and Gram-negative ones using conventional biochemical reactions (acid production from glucose and lactose, and indole, urease and H_2_S tests).

If more than 90 % of the colonies on a given plate were identical, they were considered as a positive and pure culture [22]. Mixed cultures of three or more microorganisms were regarded as contaminations and recorded as a negative growth result [21, 23]. Non-pathogenic bacteria were recorded only if isolated in pure culture [5].

Formalin-fixed biopsies were cut into 4 µm sections and stained with eosin and hematoxylin. Evaluation was performed under light microscopy for infiltration by PMN and eosinophils of the endometrial luminal epithelium and the *stratum compactum.* Samples were considered positive for acute endometritis if three or more PMN per five fields (400× magnification, BX53 optical microscope Olympus, Tokyo, Japan) were found in at least one of the biopsies [5, 6, 24] and we also evaluated the infiltration of the *stratum compactum* and *stratum spongiosum* by eosinophils using similar criteria.

The concentrations of SAA were assessed using the Multispecies SAA ELISA Kit (Tridelta Development, TP 802, Ireland) according to the manufacturer’s instructions. All samples were analyzed in duplicate. The absorbance was read at a wavelength of 450 nm using 630 nm as a reference with a Biotek μQuant reader (BioTek Instruments Inc., Winooski, VT, USA). The intra- and inter-assay CVs were 2.1 and 5.2 %, respectively. The concentration of Hp was determined by the guaiacol method according to Jones and Mould [25]. The intra-assay CV was 10.4 % and the inter-assay CV was 13.5 %.

### Statistical analysis

For statistical analysis, we used Fisher’s Exact test and the Chi square test. The significance level was set at *P* < 0.05. Sensitivity and specificity were calculated using the PMN infiltration in endometrial biopsies as the ‘gold standard’. Sensitivity was calculated as the proportion of mares with PMN infiltration in biopsies and a positive result from the compared tests (CS and UL). Specificity was calculated as the proportion of mares with no PMN infiltration of the endometrium and a negative result from the compared tests (CS and UL). A positive predictive value was calculated from the proportion of mares with PMN infiltration of the endometrium among the positive results in the compared tests (CS and UL). A negative predictive value was calculated as the proportion of mares with negative histology among the negative results in the compared tests (CS and UL) [23]. Cohen’s coefficient (*k)* was calculated to evaluate the agreement of PMN infiltration in biopsy samples taken from the left and the right horn and the positive results of cytology and bacteriology from samples obtained by CS and UL as well as changes in the appearance of the efflux. Agreement was designated as follows: poor if k ≤ 0.2, fair if 0.21 < k < 0.41, moderate if 0.41 < k < 0.6, substantial if 0.61 < k < 0.8; and good if k > 0.8 [11, 26].

## Results

### Histopathology

A total of 106 endometrial biopsies obtained from two locations were examined and PMN infiltration of the endometrium was present in 25 of 53 mares (47.2 %). Only 10 of the 25 mares with PMN infiltration of the endometrium (40 %) exhibited an increase of PMN in both biopsies that were collected from the base of each horn. Fifteen mares (60 %) had infiltration of PMN in only one horn (12 mares—left horn, 3 mares—right horn). We also observed that 25 of the subfertile mares had an eosinophil infiltration (three or more eosinophils per five high magnification fields at ×400) in at least one biopsy but only in 14 mares was this correlated with PMN infiltration of endometrium. Eleven mares had eosinophilic infiltration of the endometrium, but without PMN infiltration.

### Cytology

Positive results of cytological examination were observed in nine of 53 samples collected by lavage (17 %) and in five of 53 obtained by swabs (9.4 %, Table 1). Six samples showing the presence of PMN on cytology slides were correlated with positive results of the bacteriological examination. We observed a statistically significant relationship between PMN infiltration of the endometrium and positive cytology in samples collected by cotton swab (*P* = 0.02) and those obtained from uterine lavage (*P* = 0.0001). The sensitivity, specificity, positive predictive values (PPV) and negative predictive values (NPV) and the agreement (*k*) between PMN infiltration of the endometrium in samples obtained from the left and right uterine horns are shown in Table 1. The agreement (*k*) between PMN infiltration and positive cytology in samples obtained from CS was fair and we did not find differences between biopsies taken from the left and right uterine horns. However, the agreement between positive cytology obtained from UL and PMN infiltration of the endometrium was moderate in biopsies taken from the right uterine horn and only fair in biopsies obtained from the left horn.

**Table 1:** Results of cytology and bacteriology in relation to PMN infiltration of the endometrium

Sampling technique		Histo+	Histo−	Total	Sens	Spec	PPV	NPV	*k* left horn	*k* right horn
CS	Cul+	6	3	9	0.24	0.893	0.667	0.568	0.192	0.204
	Cul−	19	25	44						
	Total	25	28	53						
UL	Cul+	5	1	6	0.20	0.964	0.833	0.574	0.131	0.315
	Cul−	20	27	47						
	Total	25	28	53						
CS	Cyt+	5	0	5	0.20	1.00	1.00	0.583	0.256	0.228
	Cyt−	20	28	48						
	Total	25	28	53						
UL	Cyt+	9	0	9	0.360	1.00	1.00	0.636	0.277	0.431
	Cyt−	16	28	44						
	Total	25	28	53						

Positive cytological result from smears => 2 % PMNs of 300 cells (×1000); positive histological results from biopsies => 3 PMNs/5 fields (×400)

*PMN* polymorphonuclear cells, *Cul*± culture positive/negative, *Cyt*± cytology positive/negative, *Histo*± histopathology positive/negative (PMN infiltration in endometrium/no PMN infiltration in the endometrium), *CS* cotton swab, *UL* uterine lavage, *Sens* sensitivity, *Spec* specificity, *PPV* positive predictive values, *NPV* negative predictive values, *k* left/right horn- agreement between the number of PMN in biopsies samples taken from the left/right horn and the positive cytology and bacteriology in the compared tests (CS and UL)

### Microbiology

Microorganisms were isolated from six of 53 uterine lavages (11.3 %) and from nine of 53 swabs (17 %, Table 1). In three mares, positive bacteriological results were confirmed in samples collected by both CS and UL: in two cases we found *Staphylococcus* spp. and in one case *E. coli*. The most frequently isolated bacteria in samples collected by endometrial swabs were β-hemolytic streptococci isolated from five of nine (55.5 %) and *Staphylococcus* spp. in three of nine (33.3 %). *E. coli* as well as *Micrococcus* spp. were isolated from one of nine uterine swabs. The most common micro-organisms isolated from uterine lavages were *Staphylococcus* spp. isolated from three of six lavages (50 %) and *E. coli* from two of six (33.3 %). These two organisms were isolated from 83.3 % of the positive flush cultures. In one lavage we isolated *Corynebacterium* spp. (one of six lavages). The presence of bacteria and its relationship to positive cytology and histology results are presented in Table 2. We observed no significant relationship between PMN infiltration of the endometrium and positive results of microbiological examination from samples obtained by CS and UL, but there was a statistically significant relationship between positive cytology and positive microbiology in samples collected by endometrial swabs (*P* = 0.03). The Cohen’s (*k*) coefficient between positive bacteriology results from samples obtained by CS and UL and PMN infiltration of the endometrium in samples obtained from both the left and right uterine horns was usually only poor or fair, and we observed differences between the biopsy samples taken from the left and right uterine horns. The sensitivity, specificity, PPV, NPV and *k* agreement are presented in Table 1.

**Table 2:** Presence of bacteria according to results of cytological examination and PMN infiltration of the endometrium

Microorganisms	Cotton swab				Uterine lavage				Total
	Cyt+	Cyt−	Histo+	Histo−	Cyt+	Cyt−	Histo+	Histo−	
*Staphylococcus spp.*	1	2	2	1	1	2	2	1	6
β-hem. *Streptococcus*	3	2	3	2	0	0	0	0	5
*Escherichia coli*	0	1	1	0	1	1	2	0	3
*Micrococcus* spp.	0	1	1	0	0	0	0	0	1
*Corynebacterium* spp.	0	0	0	0	1	0	1	0	1

*Cyt*+/- cytology positive/negative, *Histo*± histopathology positive/negative, *PMN* polymorphonuclear cells

Relationships among

(i) macroscopic evaluation of the efflux and
(ii) PMN infiltration of the endometrium, and
(iii) positive results of cytological and bacteriological examination.

The collected lavage fluid was assessed for its volume, color and transparency as described earlier. On average we recovered 960 ± 38 ml of fluid. We observed a statistically significant relationship between infiltration of the endometrium by PMN and cloudy efflux (*P* = 0.031), but there were no statistically significant relationships between clear efflux, efflux with blood or with debris and the presence of PMN in the endometrial biopsies. The sensitivity, specificity, positive and negative predictive values (PPV and NPV) and agreement (*k)* between the infiltration of PMN in endometrial biopsies taken from the left and right uterine horns are presented in Table 3.

**Table 3:** Diagnostic evaluation of the efflux

Efflux	Sensitivity	Specificity	PPV	NPV	*k* left horn	*k* right horn
Clear	0.160	0.643	0.286	0.462	Negative values	
Cloudy	0.440	0.857	0.733	0.632	0.307	0.322
With debris	0.680	0.607	0.607	0.680	0.327	0.156
With blood	0.400	0.536	0.435	0.500	Negative values	

Sensitivity, specificity, positive and negative predictive values (PPV and NPV) and the agreement (*k*) between the number of polymorphonuclear cells (PMN) infiltrated into the endometrial luminal epithelium and *stratum compactum* and type of efflux

### Acute phase proteins (serum amyloid A and haptoglobin)

**Fig. 1:**
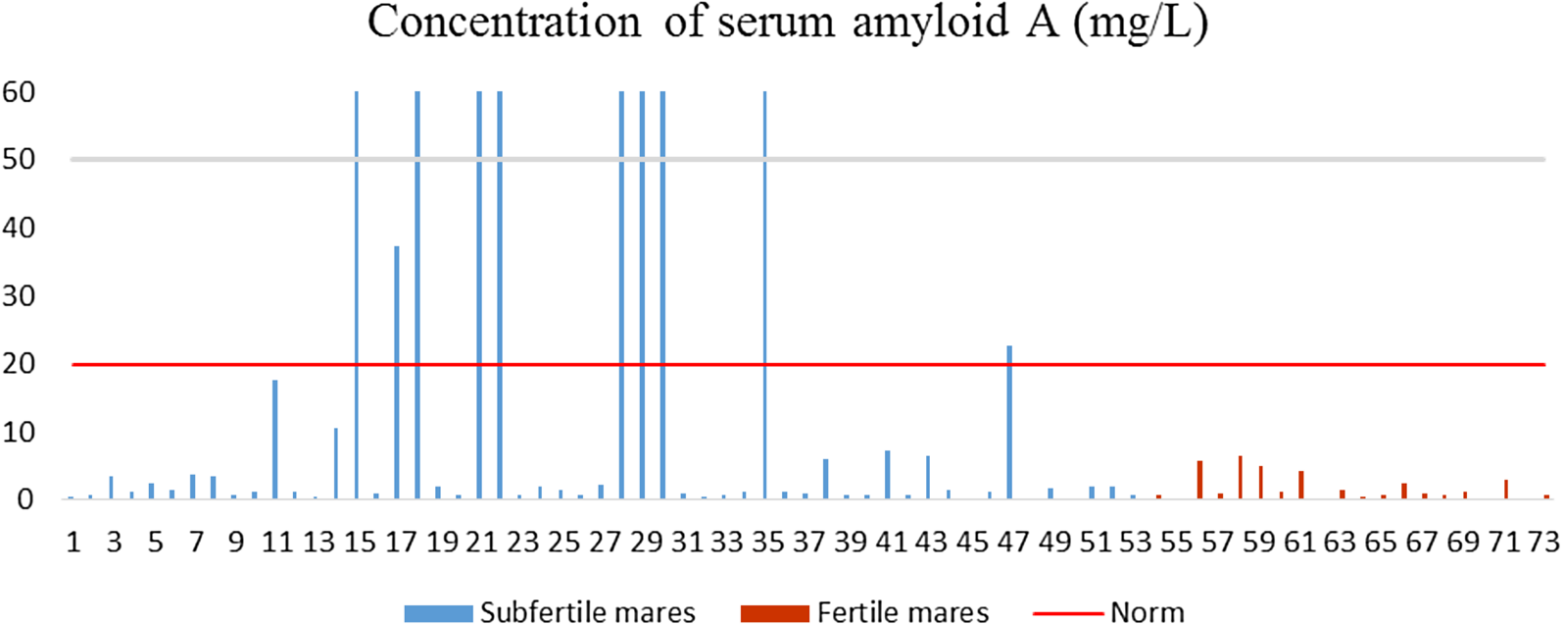
The concentration of SAA in the subfertile and fertile mares. The subfertile mares are numbered from 1 to 53 and the fertile mares are numbered from 54 to 73. Permissible norm of serum amyloid A (SAA) is from 0.5 to 20 mg/l

SAA concentrations are shown in Fig. 1. In the subfertile group, the level of SAA varied widely, and in nine mares it reached very high concentrations (from 37.26 to 595.71 mg/l), whereas in the other subfertile mares it did not exceed 20 mg/l.

**Fig. 2:**
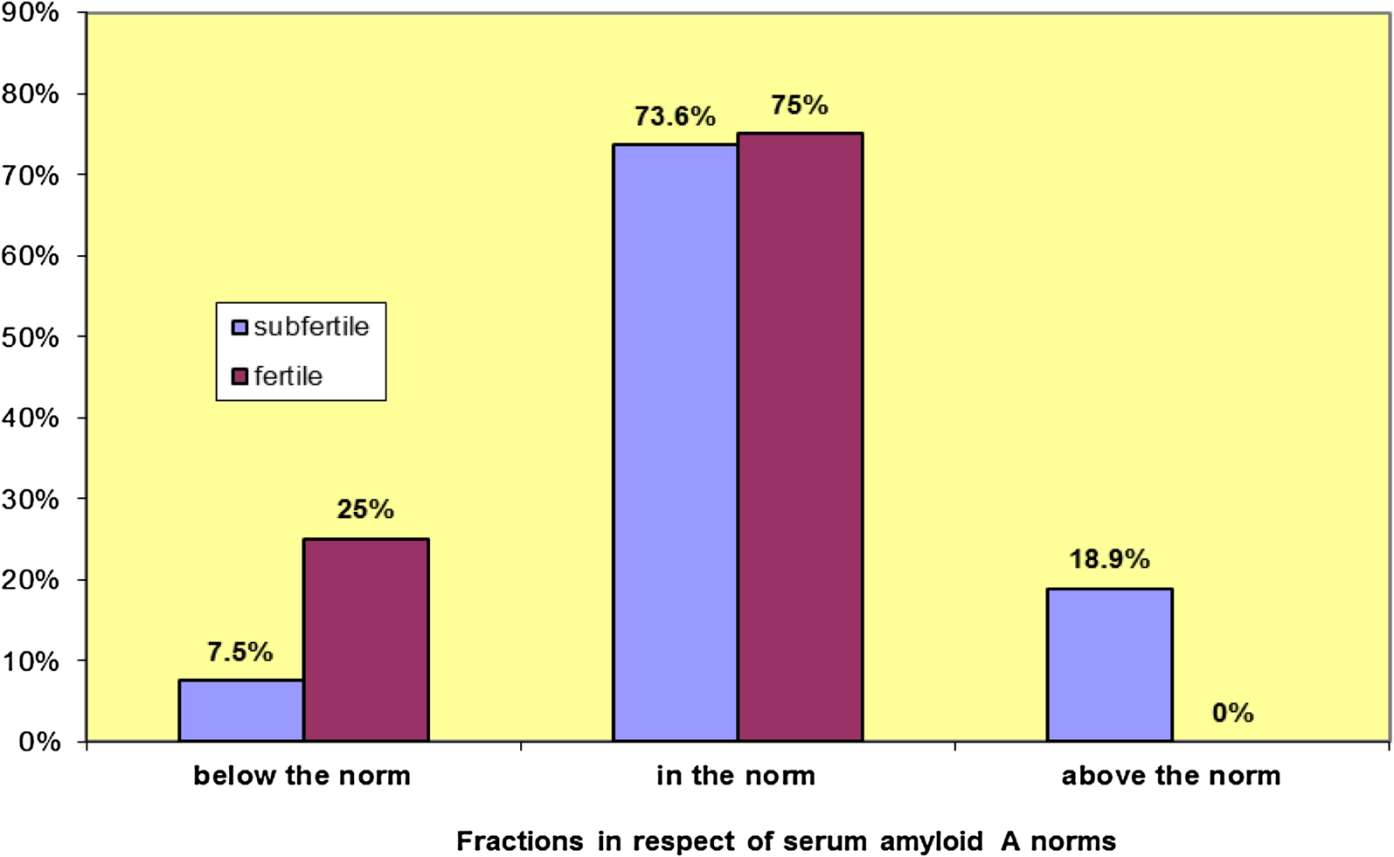
Fractions in respect of serum amyloid A norms. Below the norm (<0.5 mg/l), within the norm (0.5–20 mg/l), or above the norm (>20 mg/l), and as a group: subfertile and fertile mares

In the healthy mares from the control group, the concentration of SAA was uniform and in most cases, its level did not exceed the limit or was almost undetectable (on average 1.85 ± 2.04 mg/l, maximum 6.49 mg/l). As Fig. 2 shows, in the subfertile group 18.9 % of samples were above the norm, compared to the fertile mares in which no cases were reported.

The concentration of Hp was more constant than SAA in both groups. The Hp concentration on average was 1.27 ± 1.046 g/l in the subfertile mares and 1.13 ± 0.58 g/l in the fertile mares; these values were not significantly different.

Twelve out of 53 subfertile mares showed positive results in the bacteriological tests, but only in three mares was this associated with increased levels of SAA and Hp (*E. coli*, *Staphylococcus* spp., *Micrococcus* spp.).

## Discussion

Uterine lavage with 1 l of fluid or more is often performed for treatment of endometritis. LeBlanc et al. [5] and Christoffersen et al. [6] used a low-volume flush for diagnosing endometritis (60 and 250 ml, respectively), but the aim of our work was to demonstrate the usefulness of flushing with 1 l as a diagnostic method for endometritis.

In the present study, the sensitivity of cultures from uterine lavage was 0.20, much lower than the sensitivity levels reported by Christoffersen et al. [6] and LeBlanc et al. [5], which were 0.75 and 0.72, respectively. The lower sensitivity of the microbiology tests in our study compared to results of those reports could be due to the high dilution by fluid used for uterine lavage in our work. The low isolation of cultures may indicate either a natural resistance of Icelandic mares to uterine infections, or the good conditions for maintenance of these horses in a country involving very limited use of antibiotics in the treatment of reproductive disorders.

Christoffersen et al. [6] used a double-guarded lavage system and compared the sensitivity of samples obtained by CS, UL and biopsy. They found that the sensitivity from lavages was much higher than that from biopsies for microbiological evaluation but much lower for cytological examination. In our study, the sensitivity of cytology (0.36) obtained from UL was higher than the sensitivity (0.08) reported by Christoffersen et al. [6] using double-guarded lavage.

The bacteria most frequently isolated from UL in the study from LeBlanc et al. [5] were *E. coli* and beta-hemolytic streptococci, however, in our study the most common micro-organisms isolated from uterine flushes were *Staphylococcus* spp. followed by *E. coli.* LeBlanc et al. [5] reported that bacteria were isolated from about 45 % of the clear efflux samples and cloudy and mucoid effluxes were highly associated with isolation of bacteria. In our study, all of the clear effluxes had negative bacteriology and we did not find a correlation between the macroscopic evaluation of the recovered fluid and the type of microorganism. However, we found statistically significant relationships between a cloudy efflux and PMN infiltration of the endometrium, positive cytology and isolation of bacteria.

LeBlanc et al. [5] considered that the flushing should be regarded as contaminated if the bacteriological evaluation was positive, cytology was negative and no PMN infiltration in the endometrial biopsies was observed. In our study, all flushes from which bacteria were isolated had clarity changes and eleven of 15 cloudy effluxes (73.3 %) were associated with PMN infiltration of the endometrium. Usually, if three or more cultures are isolated, the sample is classified as having contamination [21, 23] and is recorded as a negative. In our study, mixed infections with beta-hemolytic *Streptococcus* and *Staphylococcus* spp. were found only in one CS. Overbeck et al. [11] compared five different sampling locations for collections with cytological brushes and observed that eight of 10 positive-scored uteri had both positive and negative locations for endometritis. When a large volume of liquid is infused into the uterus during lavage, it fills the entire interior of the uterus and becomes exposed to a larger surface area. Although we isolated more bacteria using CS than with lavages, the sensitivity of cytology from samples using UL was higher than from CS. Riddle et al. [27] reported that endometrial cytology identified twice as many mares with endometritis as did endometrial culture. In turn, Nielsen et al. [22] who compared cytological and bacteriological examination from two different laboratories, pointed out the importance of performing both bacteriological and cytological evaluation in diagnosis of endometritis. Moreover, Kozdrowski et al. [20] reported that the method used for cytological evaluation influences the results, and using criteria based on counting the numbers of PMN in relation to epithelial cells they found that the best cut-off point indicating endometritis is 2 % of PMN. The same criteria were also used by Overbeck et al. [11, 21].

Infiltration by PMN into the luminal epithelium and *stratum compactum* is used as the reference standard for diagnosis of endometritis [4, 5, 11, 20, 21, 23, 28]. Nielsen et al. [28] found that the infiltration of PMN into the endometrium was correlated with the 70-day pregnancy rates, in contrast to cytological evaluation, which was not correlated. Routinely in practice, only one biopsy sample is collected. Fiala et al. [10] in their study performed after slaughter concluded that only one biopsy is not enough to verify the quantity of degenerative changes in the uterus. Overbeck et al. [11], in a post mortem study using cytological brushes and endometrial biopsies, observed that the inflammation process is not the same in different uterine locations. In the present study, all of the mares were in estrus and only 10 of 25 mares with positive histology (40 %) showed increased PMN in both biopsies. Thus, fifteen of 25 mares with PMN infiltration (60 %) would not have been diagnosed as positive for endometritis if only one biopsy had been taken. The present authors agree with the opinion of Overbeck et al. [11] that it is critical to take more than one sample under clinical conditions.

Endometrial infiltration of eosinophils was found in biopsies of 25 infertile mares. Blumenthal et al. [29] in their study on endometriosis in women showed that eosinophils are involved in tissue remodeling and wound healing. They also pointed out that fibrosis is associated with eosinophil degranulation. Several older studies [30–33] affirmed that eosinophils are involved in tissue remodeling in the uterus of estrogen-treated rats and mice. Adegboyega et al. [34] observed a relationship between eosinophils and chronic endometritis in women. The authors showed that 73 % of endometrial biopsies with eosinophil infiltration should have been diagnosed as chronic endometritis, because immunohistochemical examination revealed the presence of plasma cells, which is consistent with chronic endometritis, but they did not find a statistically significant correlation between the number of eosinophils and the presence of plasma cells. In our opinion, eosinophilic infiltration is more in line with the assumptions from the studies of Adegboyega et al. [34] and Blumenthal et al. [29] than with the estrogen hypothesis, because all of the mares in our study were in estrus and only a few had increased eosinophils in the endometrium. The lack of control biopsies from fertile mares precludes us from explaining if the cycle stage and inflammation of the uterus have an influence on eosinophil infiltration in the mare endometrium.

In a study involving microscopic examination of endometrial biopsies from retired sports mares, Kilgenstein et al. [35] found only 3 % of 189 mares exhibited eosinophilic endometritis. Wehrend et al. [36] studied the distribution of eosinophils and mast cells in cervical tissue in mares and observed that eosinophils are present in the *lamina propria mucosa* and *tunica muscularis*. The authors concluded that eosinophils are part of the local cellular immune system of the equine cervix. We think that eosinophils may perform similar functions in the uterus as in the cervix. Kenney [37] and Schiemann et al. [38] suspected that pneumovagina and some other irritation causes infiltration by eosinophils. However, abnormal formation of the vulva was not detected in mares in our study, so in our opinion, the presence of eosinophils in the mare endometrium is not always connected with pneumovagina or with irritation of the endometrium.

The utility of APP as systemic markers of inflammation in horses has been studied by many authors, in response to EED, in mares with experimentally induced ascending placentitis or influenza, or as prognostic indicators for horses with inflammatory disease [17, 39–41]. Jacobsen and Andersen [14] described diseases and disease states accompanied by increased blood levels of SAA in horses. In our study, SAA and Hp levels in blood samples were examined as a potential indicator of uterine health in mares with subclinical endometritis. Krakowski et al. [17] suggested that high levels of APP already present before ovulation may be an indication of infertility in mares and could be used as an early indicator of subclinical endometritis. In contrast to the above mentioned studies, Tuppits et al. [15] did not find differences in SAA, Hp and Fb concentrations over time before and after artificial insemination. Christoffersen et al. [1] showed that plasma concentration of SAA in response to intrauterine inoculation of *E. coli* appears to be dose-dependent. Moreover, Christoffersen et al. [16] observed that during diestrus all experimental mares had increased SAA in blood samples 24 h post inoculation (pi). In our study, *E. coli* was isolated from two mares but only one of these mares showed a large increase in SAA. Tuppits et al. [15] hypothesized that the ability of local inflammation in the uterus to cause a systemic APP reaction depends on the estrous cycle stage during the inflammation and on the influence of progesterone and estrogens. The different results of these studies may depend on different cycle stages of the mares or different post-inoculation times after which blood samples were collected. Brodzki et al. [42] found that levels of SAA and Hp were higher in cows with subclinical endometritis. Nash et al. [43] found no increase of SAA 24 h after treatment but there was a high level of SAA in two cases both before and after treatment, which may be an indicator for another ongoing disease in the mares.

In our study, the concentrations of SAA and Hp were measured in blood samples collected once from 12 to 24 h after ovulation in subfertile and fertile mares and we did not find an association between APP concentrations and infertility of mares. Just as Nash et al. [43] described, we also found high concentrations of SAA in a few mares, which may perhaps suggest another inflammatory process occurring in the mares that may or may not indirectly affect infertility.

## Conclusions

The usefulness of SAA and Hp concentrations as a markers for subclinical endometritis in mares could not be conclusively demonstrated. The high concentrations found in a few cases were not associated with subclinical endometritis. Probably, they are an indication of another ongoing disease in the mares.

The association of cloudy lavage efflux and isolation of bacteria may point to a practical aspect of uterine lavage. While changes in the fluids collected are not always indicative of infection, they can indicate inflammatory changes occurring within the uterus with a high probability. We conclude that if cloudy efflux or debris is seen in the lavage fluid, when 1 l of fluid is used, the mare has endometritis and requires treatment.

## Abbreviations

APP: acute phase proteins
CS: cotton swab
Cul±: microbiology positive/negative
CV: coefficient of variation
Cyt±: cytology positive/negative
EB: endometrial biopsy
EED: early embryonic death
Fb: fibrinogen
Histo±: histopathology positive/negative
Hp: haptoglobin
*k*: Cohen’s coefficient agreement
NPV: negative predictive values
pi: post inoculation
PMN: polymorphonuclear cells
PPV: positive predictive values
SAA: serum amyloid A
Sens: sensitivity
Spec: specificity
UL: uterine lavage

## References

[1] Christoffersen M, Woodward E, Bojesen AM, Jacobsen S, Petersen MR, Troedsson MHT, Lehn-Jensen H (2012). Inflammatory responses to induced infectious endometritis in mares resistant or susceptible to persistent endometritis. BMC Vet Res.

[2] Traub-Dargatz JL, Salman MD, Voss JL (1991). Medical problems of adult horses as ranked by equine practitioners. J Am Vet Res.

[3] LeBlanc MM, Causey RC (2009). Clinical and subclinical endometritis in the mare: both threats to fertility. Reprod Domest Anim.

[4] Buczkowska J, Kozdrowski R, Nowak M, Raś A, Staroniewicz Z, Siemieniuch MJ (2014). Comparison of the biopsy and cytobrush techniques for diagnosis of subclinical endometritis in mares. Reprod Biol Endocrinol.

[5] LeBlanc MM, Magsig J, Stromberg AJ (2007). Use of a low-volume uterine flush for diagnosing endometritis in chronically infertile mares. Theriogenology.

[6] Christoffersen M, Brandis L, Samuelsson J, Bojesen AM, Troedsson MHT, Petersen MR (2015). Diagnostic double-guarded low-volume uterine lavage in mares. Theriogenology.

[7] Schoon HA, Schoon D, Klug E (1997). Die Endometriumbiopsie bei der Stute im klinisch-gynaekologischen Kontext. Pferdeheilkunde.

[8] Ricketts SW, Barrelet A (1997). A retrospective review of the histopathological features seen in a series of 4241 endometrial biopsy samples collected from UK Thoroughbred mares over a 25 year period. Pferdeheilkunde.

[9] Snider TA, Sepoy C, Holyoak GR (2011). Equine endometrial biopsy reviewed: observation, interpretation and application of histopathologic data. Theriogenology.

[10] Fiala SM, Esmeraldino A, Jobim MIM, Garbade P, Wolf CA, Richter G, Gregory RM, Mattos RC (2010). Endometrial fibrotic changes. Is one biopsy enough to diagnose degenerative changes?. Anim Reprod Sci..

[11] Overbeck W, Jäger K, Schoon H-A, Witte TS (2013). Comparison of cytological and histological examinations in different locations of the equine uterus- an in vitro study. Theriogenology.

[12] Cray C, Belgrave RL (2014). Haptoglobin quantitation in serum samples from clinically normal and clinically abnormal horses. J Equine Vet Sci.

[13] Crisman MV, Scarratt WK, Zimmerman KL (2008). Blood proteins and inflammation in the horse. Vet Clin North Am Equine Pract.

[14] Jacobsen S, Andersen PH (2007). The acute phase protein serum amyloid A (SAA) as a marker of inflammation in horses. Equine Vet Educ.

[15] Tuppits U, Orro T, Einarsson S, Kask K, Kavak A (2014). Influence of the uterine inflammatory response after insemination with frozen-thawed semen on serum concentrations of acute phase proteins in mares. Anim Reprod Sci.

[16] Christoffersen M, Baagoe CD, Jacobsen S, Bojesen AM, Petersen MR, Lehn-Jensen H (2010). Evaluation of the systemic acute phase response and endometrial gene expression of serum amyloid A and pro- and anti-inflammatory cytokines in mares with experimentally induced endometritis. Vet Immunol Immunop.

[17] Krakowski L, Krawczyk CH, Kostro K, Stefaniak T, Novotny F, Obara J (2011). Serum levels of acute phase proteins: SAA, Hp and progesterone (P4) in mares with early embryonic death. Reprod Domest Anim.

[18] Walter J, Neuberg KP, Failing K, Wehrend A (2012). Cytological diagnosis of endometritis in the mare: investigations of sampling techniques and relation to bacteriological results. Anim Reprod Sci.

[19] Ball BA, Shin SJ, Pattern VH, Lein DH, Woods GL (1988). Use of a low-volume uterine flush for microbiologic and cytologic examination of the mare’s endometrium. Theriogenology.

[20] Kozdrowski R, Sikora M, Buczkowska J, Nowak M, Raś A, Dzięcioł M (2015). Effects of cycle stage and sampling procedure on interpretation of endometrial cytology in mares. Anim Reprod Sci..

[21] Overbeck W, Witte TS, Heuwieser W (2011). Comparison of three diagnostic methods to identify subclinical endometritis in mares. Theriogenology.

[22] Nielsen JM, Troedsson MH, Pedersen MR, Bojesen AM, Lehn-Jensen H, Zent WW (2010). Diagnosis of endometritis in the mare based on bacteriological and cytological examinations of the endometrium: comparison of results obtained by swabs and biopsies. J Equine Vet Sci.

[23] Nielsen JM (2005). Endometritis in the mare: a diagnostic study comparing cultures from swab and biopsy. Theriogenology.

[24] Ricketts SW, Alonso S (1991). Assessment of the breeding prognosis of mares using paired endometrial biopsy techniques. Equine Vet J.

[25] Jones GE, Mould DL (1984). Adaptation of Guaiacol (peroxidase) test for haptoglobins to microtitration plate system. Res Vet Sci.

[26] Cocchia N, Paciello O, Auletta L, Uccello V, Silvestro L, Mallardo K, Paraggio G, Pasolini MP (2012). Comparison of cytobrush, cotton swab, and low-volume uterine flush techniques to evaluate endometrial cytology for diagnosing endometritis in chronically infertile mares. Theriogenology.

[27] Riddle WT, LeBlanc MM, Stromberg AJ (2007). Relationships between uterine culture, cytology and pregnancy rates in a Thoroughbred practice. Theriogenology.

[28] Nielsen JM, Nielsen FH, Peersen MR (2012). Diagnosis of equine endometritis—microbiology, cytology and histology of endometrial biopsies and correlation to fertility. Pferdeheilkunde.

[29] Blumenthal RD, Samoszuk M, Taylor AP, Brown G, Alisauskas R, Goldenberg DM (2000). Degranulating eosinophils in human endometriosis. Am J Pathol.

[30] Brokelmann J, Fawcett D (1969). The localization of endogenous peroxidase in the rate uterus and its induction by estradiol. Biol Reprod.

[31] Bassett E (1962). Infiltration of eosinophils into the modified connective tissue of oestrus and pregnant animals. Nature.

[32] Bergman F, Damber M, Linden U, Paul K (1972). Mast cells and eosinophil granulocytes in the oestrogen-stimulated mouse uterus. Acta Endocrinol.

[33] King W, Alen T, DeSombre E (1981). Localization of uterine peroxidase activity in estrogen-treated rats. Biol Reprod.

[34] Adegboyega PA, Pei Y, McLarty J (2012). Relationship between eosinophils and chronic endometritis. Hum Pathol.

[35] Kilgenstein HJ, Schoeniger S, Schoon D, Schoon HA (2015). Microscopic examination of endometrial biopsies of retired sports mares: an explanation for the clinically observed subfertility?. Res Vet Sci.

[36] Wehrend A, Huchzermeyer S, Bostedt H (2005). Distribution of eosinophils and mast cells in the cervical tissue of non-gravid mares during dioestrus. Reprod Dom Anim.

[37] Kenney RM (1978). Cyclic and pathologic changes of the mare endometrium as detected by biopsy, with a note on early embryonic death. J Am Vet Med Assoc.

[38] Schiemann V, Bartmann CP, Kirpal G, von Reiswitz A, Schoon H-A, Klug E (2001). Diagnostic hysteroscopy in the mare- uterine contamination and endometrial reaction. Pferdeheilkunde.

[39] Coutinho da Silva MA, Canisso IF, MacPherson ML, Johnson AEM, Divers TJ (2013). Serum amyloid A concentration in healthy periparturient mares and mares with ascending placentitis. Equine Vet J.

[40] Hulten C, Sandgren B, Skioldebrand E, Klingeborn B, Marhaug G, Forsberg M (1999). The acute phase protein serum amyloid A (SAA) as an inflammatory marker in equine influenza virus infection. Acta Vet Scand.

[41] Westerman TL, Tornquist SJ, Foster CM, Poulsen KP (2015). Evaluation of serum amyloid A and haptoglobin concentrations as prognostic indicators for horses with inflammatory disease examined at a tertiary care hospital. Am J Vet Res.

[42] Brodzki P, Kostro K, Krakowski L, Marczuk J (2015). Inflammatory cytokine and acute phase protein concentrations in the peripheral blood and uterine washings of cows with subclinical endometritis in the late postpartum period. Vet Res Commun.

[43] Nash DM, Sheldon IM, Herath S, Lane EA (2010). Markers of uterine innate immune response of the mare. Anim Reprod Sci.

